# Biochemical and antioxidant responses of common carp (*Cyprinus carpio*) exposed to sublethal concentrations of the antiepileptic and analgesic drug gabapentin

**DOI:** 10.17221/75/2024-VETMED

**Published:** 2025-01-20

**Authors:** Jana Blahova, Premysl Mikula, Petr Marsalek, Zdenka Svobodova

**Affiliations:** Department of Animal Protection and Welfare and Veterinary Public Health, Faculty of Veterinary Hygiene and Ecology, University of Veterinary Sciences Brno, Brno, Czech Republic

**Keywords:** enzyme activities, fish, oxidative stress, pharmaceuticals

## Abstract

The present study aimed to evaluate the biochemical and antioxidant responses of common carp after exposure to the antiepileptic and analgesic drug gabapentin at concentrations of 0.1, 1, 10, and 100 μg/l for 4 weeks. The exposure to the highest two concentrations resulted in significant changes in plasma indices such as glucose (only group 100 μg/l), alanine aminotransferase (ALT), lactate dehydrogenase (LDH), lipase, creatine kinase (CK), amylase as well as butyrylcholinesterase (BChE). Similar trends were found in both groups exposed to the environmentally relevant concentrations (i.e., 0.1 and 1 μg/l). In addition, a significant increase in the ferric-reducing power of the plasma was noted in all treated groups. Numerous changes in antioxidant enzymes, superoxide dismutase, catalase, glutathione-S-transferase, as well as lipid peroxidation, were observed especially in the caudal kidney in the group exposed to 100 μg/l. Significant findings were also confirmed in the group exposed to an environmentally relevant concentration (1 μg/l), with a decrease in superoxide dismutase in the gill and an increased lipoperoxidation in the caudal kidney. Our research shows that subchronic exposure to gabapentin may pose a significant risk to non-target aquatic organisms, such as disruption of metabolic pathways or induction of oxidative stress, even at environmentally relevant concentrations.

Pharmaceuticals are a major group of emerging environmental pollutants and present a new global water quality challenge. They enter the freshwater and marine environment due to intensive anthropogenic activities. The human consumption of pharmaceuticals has been significantly growing over the last few years, not only due to the increase in population and aging but mainly due to current lifestyles (Garg et al. 2023).

Gabapentin is an important structural analogue of the most abundant inhibitory neurotransmitter, γ-aminobutyric acid (GABA). In human therapy, it is mainly used as an effective anticonvulsant for the treatment of epilepsy, but it can also reduce neuropathic pain or uremic pruritus. Its consumption has been increasing significantly in recent years. According to the data from the State Institute for Drug Control of the Czech Republic, annual gabapentin consumption in 2011 was 8 420 kg, whereas in 2020, it was already 15 390 kg (Ferencik et al. 2022). As this substance is usually not sufficiently eliminated by wastewater treatment processes, it can enter the aquatic ecosystems, presenting a potential threat to non-target aquatic organisms (Gurke et al. 2015). Ferencik et al. (2022) monitored the occurrence of selected antiepileptic drugs in the Elbe River, and gabapentin was found to be the most frequently occurring representative. At the sites monitored, its concentrations in water reached a few hundred ng/l. Residues of this substance are also found in surface waters abroad, with a very significant prevalence at relatively high concentrations (Gurke et al. 2015). Detected concentrations often reached thousands of ng/l (Henning et al. 2018). Quite alarming are also the findings of gabapentin in raw drinking water in Switzerland at average concentrations of tens of ng/l (Morasch et al. 2010). Among many other human and veterinary therapeutics monitored, considering the mean concentration of its residues in wastewater treatment plant effluents, gabapentin was found to be the fourth most important pharmaceutical in the Baltic Sea region, with a maximum concentration reaching 10 μg/l (UNESCO and HELCOM 2017).

Although gabapentin consumption is on the rise and its occurrence in aquatic environments is widespread, there is a lack of scientific data addressing its toxicity to fish. Given acute toxicity, gabapentin poses no risk to aquatic organisms (Li et al. 2018). On the other hand, chronic exposure of aquatic organisms to even low concentrations may already be a significant risk (Salahinejad et al. 2023). Li et al. (2018) documented that gabapentin exposure exerted developmental toxicity and led to oxidant injury in the early life stages of zebrafish. They also confirmed the neuro- and immunotoxicity of gabapentin to zebrafish embryos at realistic environmental concentrations (He et al. 2019). Further, O’Rourke et al. (2023) reported strong perturbations of the central carbon metabolism in daphnids after sub-chronic exposure to gabapentin.

As mentioned above, there are only a few *in vivo* ecotoxicological reports on gabapentin toxicity. However, from the data available, it is clear that gabapentin may pose a significant health risk to fish, particularly with chronic exposure. Therefore, we conducted an experiment to evaluate the biochemical and antioxidant responses of common carp (*Cyprinus carpio*) after a long-term (4 weeks) exposure to gabapentin. The common carp was chosen as a model species for our study because it is a common freshwater fish species living in our waters and plays an important role in aquaculture in the Czech Republic.

## MATERIAL AND METHODS

### Experimental design

A total of 120 juveniles of common carp (*C. carpio*) obtained from the Mendel University Brno (Czech Republic) with a mean weight of 50.0 ± 1.6 g were included in the toxicity test. Fish were randomly distributed into ten 200 l aquaria with dechlorinated tap water and continuous aeration (i.e., 12 fish in each aquarium) connected to a flow-through system. Before the toxicity test started, the fish were acclimatised to laboratory conditions for 2 weeks. After the adaptation period, fish with a mean weight of 62.1 ± 3.4 g were exposed to four concentrations of gabapentin in water (0.1, 1, 10 and 100 μg/l) for four weeks. The test chemical gabapentin (CAS 60142-96-3; chemical purity ≥ 99%) in powder form was purchased from Sigma-Aldrich Chemical Co. (St. Louis, USA). To prepare the required concentrations, a stock solution of gabapentin (80 mg/l) was dosed into the aquarium water in the appropriate volume. A stock solution was prepared twice weekly and stored in a refrigerator at 4 °C. To verify the real concentration of the test substance in each aquarium, water samples from all experimental groups were taken twice a week during the whole trial. Water samples were stored in a deep freezer (–80 °C) until analysis using LC/MS. The lowest two concentrations tested corresponded to environmentally relevant concentrations of gabapentin detected in surface waters in the Czech Republic (Ferencik et al. 2022). Higher concentrations were chosen as multiples of the lower ones to assess the dose-response effects. The control group was also included in the trial. All groups were performed in duplicate. The fish were fed a commercial feed (Skretting 3 mm; Fontaine-les-Vervins, France) three times a day at a rate of 3% of body weight per day. Biweekly, the fish were reweighted and the feeding dose was adjusted. Unconsumed feed and excrement from each aquarium were removed daily using a fish net, and the bottoms and internal surfaces were carefully cleaned. The daily photoperiod was set to 12 h of light and 12 h of dark. The basic physicochemical characteristics of the water in all the aquaria were monitored daily. While the temperature during the test ranged from 21.6 to 22.9 °C, dissolved oxygen did not fall below 70%, and pH values were kept between 7.45 and 8.22. The health condition of the fish was checked daily.

The toxicity test was conducted in the approved facility of the Department of Animal Protection and Welfare and Veterinary Public Health (University of Veterinary Sciences Brno, Czech Republic) in strict compliance with the relevant national legislation (Act No. 246/1992 Coll., Decree No. 419/2012 Coll.). The testing was approved by the Ministry of Education, Youth, and Sports of the Czech Republic; the number of the approved experimental project was MSMT-12385/2023-3.

### Verification of gabapentin in water

Water samples were filtered through a 0.22 μm nylon filter (Millipore, Burlington, USA) and used for LC/MS analysis. A Thermo Scientific UHPLC Accela 1250 system was connected to a Thermo Scientific TSQ Quantum Access MAX Triple Quadrupole Instrument (Thermo Scientific, Waltham, USA) equipped with a heated electrospray ionization probe. The instrument was calibrated daily with multi-level calibration curves for our quality assurance and quality control program. Procedural blank and solvent blank were analysed for every set of 10 samples. The inter-day precision expressed as a relative standard deviation was 9.1%. The current concentrations of gabapentin in water in the experimental groups were as follows: 0.11 ± 0.0, 1.0 ± 0.1, 10.8 ± 0.5, and 113.4 ± 4.9 μg/l. The concentration of gabapentin in the control group was below the limit of detection (0.071 μg/l).

### Blood and tissue sampling

After four weeks of exposure to gabapentin, fish were subjected to blood sampling. Blood was withdrawn from the caudal vein by a heparinized syringe and collected into tubes to prepare plasma for the analysis of biochemical and oxidative stress indices. The fish were stunned with a blow to the head, bled to death by cutting their gill arches and submitted to morphometric analysis (body and total body length, body and liver weight, hepatosomatic index – HSI, and Fulton’s condition factor – FCF) and autopsy. The Fulton’s condition factor (FCF) was calculated as body weight in g/(standard length in cm)^3^. At autopsy, liver, gill, and caudal kidney samples were collected for the analysis of antioxidant enzyme activities and tissue lipoperoxidation. All samples were stored immediately after collection in a deep freezer (–80 °C) until analysis.

### Plasma biochemical indices

Plasma was used for the analysis of basic biochemical parameters including indices of carbohydrate (glucose, lactate), lipid (triacylglycerols, cholesterol), and nitrogen (albumin, total protein, ammonia, creatinine) metabolism. In addition, the concentration of minerals (chloride, phosphorus, magnesium, calcium, iron) and the activities of selected enzymes (alkaline phosphatase – ALP, alanine aminotransferase – ALT, aspartate aminotransferase – AST, lactate dehydrogenase – LDH, lipase, creatine kinase – CK, amylase, butyrylcholinesterase – BCheE) were monitored. Commercial kits from Biovendor (Brno, Czech Republic) and the Konelab 20i biochemical analyser were used for the determination. Oxidative stress markers were also evaluated in plasma, namely the ferric-reducing power of plasma (FRAP) and ceruloplasmin activity. A detailed description of the methods used is described by Haluzova et al. (2010).

### Antioxidant enzyme activities and tissue lipoperoxidation

The assessment of oxidative stress was performed in the liver, gill, and caudal kidney tissue, specifically the analysis of antioxidant and detoxifying enzymes (superoxide dismutase – SOD, catalase – CAT, glutathione peroxidase – GPx, glutathione reductase – GR and glutathione-S-transferase – GST) and the evaluation of lipoperoxidation rate measured by TBARS assay (thiobarbituric acid reactive substances). Before the analysis, tissue samples were first homogenised in phosphate buffer. All analyses were performed spectrophotometrically. Enzyme activities were converted to protein content. A detailed description of the methods used is given in our previous study (Mikula et al. 2024).

### Data analysis

Statistical data processing was performed in Unistat v6.5 for Excel (Unistat Ltd, UK). First, normality testing (Shapiro-Wilk test) and homogeneity of variance (Levene’s test) were performed. When the normal distribution condition was met, one-factor analysis of variance (ANOVA) and post hoc Tukey-HSD test were used. When the normality condition was not met, the multiple median test was used. Testing was performed at a significance level of *P* < 0.05.

## RESULTS

### Mortality, behaviour, and morphometric indices

No mortality and behavioural changes were recorded during the acclimatization period as well as during the four-week exposure.

Table 1 shows the results of morphometric indices. Data analysis did not confirm statistically significant differences between groups for any studied variables (*P* > 0.05).

**Table 1:** Morphometric indices in common carp after 4-week exposure to gabapentin (GAB)

Indices	Control	GAB 0.1 μg/l	GAB 1 μg/l	GAB 10 μg/l	GAB 100 μg/l
Total length (mm)	164.2** ± **2.9	158.4** ± **2.7	160.3** ± **2.3	164.1** ± **2.1	160.5** ± **2.2
Standard length (mm)	143.9** ± **2.6	139.5** ± **2.3	140.4** ± **1.9	144.7** ± **2.0	141.3** ± **1.8
Body weight (g)	94.1** ± **5.8	81.7** ± **5.2	81.1** ± **4.1	92.4** ± **4.7	83.8** ± **3.7
Liver weight (g)	2.9** ± **0.2	2.5** ± **0.2	2.5** ± **0.2	2.6** ± **0.2	2.5** ± **0.2
HSI	3.0** ± **0.1	3.1** ± **0.1	3.0** ± **0.2	2.8** ± **0.1	3.1** ± **0.2
FCF	3.0** ± **0.1	2.9** ± **0.0	2. 9** ± **0.0	3.0** ± **0.0	2.9** ± **0.0

Data are given as the mean ± standard error of the mean. No significant differences (*P* > 0.05) were found among groups

FCF = Fulton’s condition factor; HSI = hepatosomatic index

### Plasma biochemical indices

Many statistically significant differences (*P *< 0.05) were observed in the biochemical examination of blood plasma compared to the control group (Tables 2 and 3). These included mainly changes in the activities of the following enzymes – ALT, LDH, CK, lipase, amylase, and BChE. The most frequent changes were in the experimental groups exposed to the highest concentrations tested (i.e., 10 and 100 μg/l). Surprisingly, there were statistically significant (*P* < 0.05) increases in LDH, CK, BChE, and lipase (only for 1 μg/l) in the experimental groups exposed to the environmentally relevant concentrations tested. There were no significant differences in the parameters of nitrogen and lipid metabolism, and the mineral profile compared to the control group. In the case of carbohydrate metabolism, there was a statistically significant increase (*P *< 0.05) in glucose compared to the control group but only in the experimental groups exposed to gabapentin at concentrations of 1 and 100 μg/l.

**Table 2:** Plasma biochemical indices in common carp after 4-week exposure to gabapentin (GAB)

Indices	Control	GAB 0.1 μg/l	GAB 1 μg/l	GAB 10 μg/l	GAB 100 μg/l
Carbohydrate metabolism					
Glucose (mmol/l)	3.9 ± 0.3^b^	5.6 ± 0.3^ab^	**6.0** ± **0.3**^a^↑	5.3 ± 0.4^ab^	**6.0** ± **0.3**^a^↑
Lactate (mmol/l)	2.2 ± 0.4^a^	3.0 ± 0.5^a^	1.7 ± 0.3^a^	3.2 ± 0.5^a^	2.3 ± 0.7^a^
Lipid metabolism					
Cholesterol (mmol/l)	5.7 ± 0.4^a^	6.3 ± 0.2^a^	6.5 ± 0.3^a^	5.8 ± 0.2^a^	6.1 ± 0.3^a^
Triacylglycerols (mmol/l)	4.0 ± 0.4^a^	4.5 ± 0.4^a^	4.9 ± 0.4^a^	4.2 ± 0.4^a^	4.8 ± 0.6^a^
Nitrogen metabolism					
Albumin (g/l)	12.9 ± 0.5^a^	13.2 ± 0.6^a^	14.3 ± 0.7^a^	13.0 ± 0.6^a^	13.4 ± 0.7^a^
Total protein (g/l)	32.3 ± 1.2^a^	33.7 ± 0.9^a^	35.6 ± 0.7^a^	32.8 ± 0.8^a^	34.7 ± 0.9^a^
Ammonia (μmol/l)	257.0 ± 22.2^a^	235.5 ± 21.0^a^	222.6 ± 16.6^a^	267.6 ± 20.3^a^	238.6 ± 17.6^a^
Creatinine (μmol/l)	18.4 ± 1.0^a^	19.4 ± 1.1^a^	18.2 ± 1.3^a^	21.7 ± 1.1^a^	18.6 ± 0.9^a^
Minerals					
Chlorides (mmol/l)	114.1 ± 0.7^a^	113.3 ± 0.5^a^	113.6 ± 1.1^a^	116.3 ± 1.0^a^	116.1 ± 1.7^a^
Phosphorus (mmol/l)	1.5 ± 0.1^a^	1.5 ± 0.1^a^	1.6 ± 0.1^a^	1.8 ± 0.1^a^	1.6 ± 0.2^a^
Magnesium (mmol/l)	0.8 ± 0.0^a^	0.9 ± 0.0^a^	0.9 ± 0.0^a^	0.9 ± 0.0^a^	0.9 ± 0.0^a^
Calcium (mmol/l)	2.5 ± 0.0^a^	2.6 ± 0.0^a^	2.5 ± 0.0^a^	2.6 ± 0.0^a^	2.6 ± 0.1^a^
Iron (mmol/l)	22.0 ± 1.7^a^	22.8 ± 2.4^a^	23.7 ± 2.1^a^	20.9 ± 1.7^a^	25.9 ± 1.8^a^

Data are given as the mean ± standard error of the mean (*n* = 10 in each group). ^a,b^Significant differences (*P* < 0.05) among groups are indicated by different alphabetical superscripts. Significantly different results compared to the control are described by bold font and an arrow indicating the direction of the regulation

**Table 3:** Plasma enzyme activities in common carp after 4-week exposure to gabapentin (GAB)

Indices	Control	GAB 0.1 μg/l	GAB 1 μg/l	GAB 10 μg/l	GAB 100 μg/l
ALP (μkat/l)	0.5 ± 0.1^a^	0.3 ± 0.0^a^	0.4 ± 0.0^a^	0.3 ± 0.0^a^	0.4 ± 0.1^a^
ALT (μkat/l)	0.3 ± 0.0^c^	0.4 ± 0.0^c^	**0.8** ± **0.1**^a^↑	**0.6** ± **0.0**^b^↑	**0.6** ± **0.0**^b^↑
AST (μkat/l)	2.8 ± 0.4^a^	2.4 ± 0.4^a^	2.3 ± 0.4^a^	2.5 ± 0.4^a^	3.2 ± 0.5^a^
Amylase (μkat/l)	0.6 ± 0.1^a^	0.4 ± 0.1^ab^	0.5 ± 0.1^ab^	**0.2** ± **0.0**^b^↓	**0.2** ± **0.1**^b^↓
CK (μkat/l)	76.1 ± 4.6^b^	**121.7** ± **11.2**^a^↑	**136.7** ± **12.2**^a^↑	**141.8** ± **14.5**^a^↑	**118.9** ± **14.2**^a^↑
BChE (μkat/l)	1.2 ± 0.1^b^	**2.0** ± **0.2**^a^↑	**2.9** ± **0.3**^a^↑	**2.4** ± **0.3**^a^↑	**2.5** ± **0.3**^a^↑
LDH (μkat/l)	7.3 ± 1.5^b^	**17.0** ± **2.0**^a^↑	**21.5** ± **2.4**^a^↑	**21.3** ± **2.2**^a^↑	**21.4** ± **2.0**^a^↑
Lipase (μkat/l)	0.2 ± 0.0^b^	0.3 ± 0.0^ab^	**0.4** ± **0.1**^b^↑	**0.5** ± **0.1**^b^↑	**0.4** ± **0.0**^b^↑

Data are given as the mean ± standard error of the mean (*n* = 10 in each group). ^a–c^Significant differences (*P* < 0.05) among groups are indicated by different alphabetical superscripts. Significantly different results compared to the control are described by bold font and an arrow indicating the direction of the regulation

ALP = alkaline phosphatase; ALT = alanine aminotransferase; AST = aspartate aminotransferase; BChE = butyrylcholinesterase; CK = creatine kinase; LDH = lactate dehydrogenase

### Oxidative stress indices

**Figure 1:**
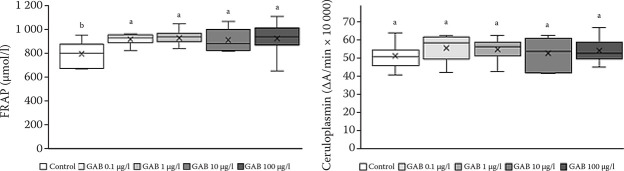
The ferric-reducing power of plasma (FRAP) and ceruloplasmin activity in the blood plasma of common carp after four-week exposure to gabapentin (GAB) Within each boxplot, horizontal lines denote median values; the cross indicates mean value, boxes extend from the 25 to the 75 percentile of each group’s distribution of value, lower and upper whiskers indicate the smallest value within the 1.5 times interquartile range below 25 and the largest value within 1.5 times interquartile range above 75 percentile, respectively. ^a,b^Significant differences (*P* < 0.05) among groups are indicated by different alphabetical superscripts

The rate of oxidative stress induction was monitored in plasma (FRAP, ceruloplasmin) and selected tissues such as the liver, gills, and caudal kidney (SOD, CAT, GPx, GR, GST, TBARS). The results are shown in Figure 1 and Table 4. Due to low activity, the GR was not analysed in the caudal kidney samples. A statistically significant (*P* < 0.05) increase in FRAP was observed in all experimental groups, including the groups exposed to environmentally relevant concentrations (i.e., 0.1 and 1 μg/l). In tissue analysis, the greatest changes were noted in the caudal kidney in the experimental group exposed to the highest concentration tested (i.e., 100 μg/l). There was a significant (*P *< 0.05) decrease in SOD, CAT, and GST activity and an increase in lipoperoxidation compared to the control group. Significant elevation (*P* < 0.05) of lipoperoxidation was also observed in almost all treated groups (except for 0.1 μg/l) in this tissue. In addition, a decrease in SOD was noted in the caudal kidney in the experimental group exposed to 10 μg/l. Further, a statistically significant elevation was documented in SOD in the liver of the experimental group exposed to the highest concentration (i.e., 100 μg/l) compared to the control group. On the other hand, the opposite trend was noted in the gill where a significant decrease (*P* < 0.05) in the SOD activity was in almost all treatment groups (except for 0.1 μg/l).

**Table 4:** Oxidative stress biomarker in the liver, gill, and caudal kidney of common carp after 4-week exposure to gabapentin (GAB)

Indices	Control	GAB 0.1 μg/l	GAB 1 μg/l	GAB 10 μg/l	GAB 100 μg/l
Liver					
SOD	9.6 ± 1.9^b^	13.7 ± 2.1^b^	11.9 ± 1.6^b^	12.2 ± 1.7^b^	**19.1** ± **2.2**^a^↑
CAT	738.1 ± 30.9^a^	737.0 ± 51.0^a^	808.5 ± 57.2^a^	817.8 ± 61.1^a^	731.8 ± 54.1^a^
GPx	152.3 ± 9.0^ab^	196.0 ± 17.1^a^	148.5 ± 8.0^b^	144.8 ± 12.0^b^	174.8 ± 10.4^ab^
GR	6.5 ± 0.5^a^	5.2 ± 0.6^a^	5.5 ± 0.5^a^	5.6 ± 0.5^a^	5.5 ± 0.3^a^
GST	206.6 ± 12.7^a^	205.1 ± 18.0^a^	214.3 ± 12.2^a^	202.3 ± 15.3^a^	204.2 ± 21.1^a^
TBARS	5.8 ± 1.5^a^	10.9 ± 1.9^a^	10.2 ± 3.0^a^	7.3 ± 2.3^a^	7.4 ± 1.9^a^
Gill					
SOD	7.4 ± 1.1^a^	4.9 ± 1.2^ab^	**3.2** ± **0.4**^b^↓	**2.1** ± **0.3**^b^↓	**3.4** ± **1.0**^b^↓
CAT	16.4 ± 0.8^a^	14.6 ± 1.1^a^	16.3 ± 1.9^a^	12.3 ± 0.8^a^	14.3 ± 0.7^a^
GPx	150.4 ± 10.1^ab^	165.9 ± 18.3^ab^	183.0 ± 7.0^a^	138.0 ± 10.5^b^	135.7 ± 8.0^b^
GR	3.1 ± 0.3^a^	2.8 ± 0.3^a^	3.1 ± 0.3^a^	2.2 ± 0.2^a^	2.8 ± 0.3^a^
GST	240.9 ± 19.4^ab^	258.7 ± 16.9^ab^	273.6 ± 20.9^a^	203.7 ± 9.1^b^	228.2 ± 15.0^ab^
TBARS	3.6 ± 1.2^a^	3.4 ± 0.7^a^	1.6 ± 0.2^a^	1.9 ± 0.3^a^	3.6 ± 0.8^a^
Caudal kidney					
SOD	13.3 ± 1.2^a^	9.0 ± 1.0^ab^	8.3 ± 1.2^ab^	**5.3** ± **0.6**^b^↓	**5.4** ± **0.7**^b^↓
CAT	99.4 ± 3.0^a^	99.7 ± 4.7^a^	91.5 ± 7.7^ab^	89.3 ± 8.1^ab^	**63.4** ± **3.8**^b^↓
GPx	318.6 ± 15.4^a^	358.0 ± 20.1^a^	354.1 ± 22.0^a^	318.2 ± 21.5^a^	349.2 ± 40.3^a^
GST	790.6 ± 29.9^a^	724.6 ± 64.0^a^	691.3 ± 21.6^a^	704.9 ± 58.9^a^	**502.9** ± **28.8**^b^↓
TBARS	1.2 ± 0.1^c^	1.8 ± 0.1^bc^	**2.7** ± **0.4**^ab^↑	**3.3** ± **0.5**^ab^↑	**2.9** ± **0.2**^a^↑

Data are given as the mean ± standard error of the mean (*n* = 10 in each group). ^a–c^Significant differences (*P* < 0.05) among groups are indicated by different alphabetical superscripts. Significantly different results compared to the control are described by bold font and an arrow indicating the direction of the regulation

CAT = catalase (μmol/min/mg protein); GPx = glutathione peroxidase (nmol/min/mg protein); GR = glutathione reductase (nmol/min/mg protein); GST = glutathione-S-transferase (nmol/min/mg protein); SOD = superoxide dismutase (U/mg protein); TBARS = thiobarbituric acid reactive substances assay for estimating lipoperoxidation (nmol/g tissue)

## DISCUSSION

The pervasive presence of pharmaceutical residues in aquatic ecosystems has raised substantial concerns regarding their potential impact on aquatic organisms. Among these contaminants, gabapentin, a widely prescribed antiepileptic and analgesic drug, has emerged as a compound of significant interest due to its frequent detection in various water bodies (Ferencik et al. 2022). The frequent occurrence of gabapentin in aquatic ecosystems raises concerns about its potential ecotoxicity. Unfortunately, there are a few scientific studies on this topic, but even in this limited body of literature, it is clear that exposure to gabapentin poses a potential risk to non-target aquatic organisms (Li et al. 2018; He et al. 2019). Furthermore, gabapentin has been newly designated as a priority substance for future analysis under the Water Framework Directive (Gomez Cortes et al. 2022).

Our study investigated the potential adverse effects of gabapentin on juvenile common carp (*C. carpio*), focusing on its impact on plasma biochemical indicators and oxidative stress indices in blood and selected tissues. The experimental design involved exposing juvenile fish to varying gabapentin concentrations, including environmentally relevant concentrations.

In our four-week toxicity test, biochemical analysis revealed significant alterations in many plasma indices, particularly in enzyme activities at higher concentrations tested. Surprisingly, some enzyme changes were also noted at environmental concentrations (i.e., 0.1 and 1 μg/l), indicating that even low levels of gabapentin can have significant physiological impacts on aquatic organisms. This underlines the importance of monitoring pharmaceutical contaminants in aquatic environments and assessing their ecological risks. The exposure to the highest two concentrations of gabapentin (i.e., 10 and 100 μg/l) led to a significant elevation of the following plasma enzymes – ALT, LDH, lipase, CK, and BChE. In general, the elevation of the mentioned enzymes usually indicates liver tissue damage, but this is not entirely consistent with the other results in our study, where no significant changes were found in ALP and AST as reliable indicators of liver function. Besides, there were no significant alterations of the lipid and nitrogen metabolism indicators, which can also be used as good indicators of liver damage (e.g., albumin, ammonia) (Tamber et al. 2023). Damage to liver tissue following exposure to various contaminants is relatively common (Mikula et al. 2024), as the liver is the main detoxifying organ, thus there are increased demands on its function. However, gabapentin is minimally biotransformed in the liver and most of it is excreted by the kidneys in unchanged form. Therefore, a higher load on this organ is expected (McLean 1994; Terry et al. 2010). Thus, it is more likely that, for example, the elevated CK activity in our study may be related to possible kidney damage as an indicator of energy and tissue impairment in fish (Baldissera and Baldisserotto 2021). Since the kidneys play a key role in excreting gabapentin, damage to this organ might manifest. This hypothesis is partly supported by the results of the oxidative stress measurements, where many significant findings in this organ were documented, as discussed below. Anyway, further research will be needed, focusing also on other biomarkers such as histological examination of tissues.

In contrast to the elevation of the mentioned plasma enzymes, amylase activity showed a statistically significant decrease at the two highest gabapentin concentrations (i.e., 10 and 100 μg/l), which is quite unexpected and requires further investigation. Amylase is a key enzyme for breaking down starches into simpler sugars, such as maltose and glucose, to make energy sources available to organisms. This can lead to reduced glucose absorption from feed in the digestive tract. The inhibition of amylase could reflect a change in the production or secretion of pancreatic enzymes, possibly because of gabapentin’s impact on endocrine function or suppression of stress-related pancreatic activity during prolonged exposure (Nolasco-Soria 2021). The confirmation that the fish were stressed after exposure to gabapentin can also be supported by the elevated glucose in the treated groups, which were tested as significant at the 1 and 100 μg/l concentrations. In general, an increased glucose concentration indicates a stress response of the organism to various endogenous and exogenous factors, including anthropogenic contaminants (Martinez-Porchas et al. 2009).

Further, our study confirmed that exposure of common carp to gabapentin induces oxidative stress even at low environmentally relevant concentrations tested. Oxidative stress represents an imbalance between the capacity of the antioxidant system and the formation of reactive oxygen and nitrogen species (Hoseinifar et al. 2021). The ability to induce oxidative stress in fish has been demonstrated for many aquatic contaminants, not only after exposure to pharmaceuticals (Mikula et al. 2024) but also pesticides (Haluzova et al. 2010), musk substances (Cahova et al. 2023), or other pollutants. In our experiment, the antioxidant capacity of plasma measured by the FRAP assay increased statistically significantly in all experimental groups, i.e. even at the lowest concentration tested. This systematic antioxidant response reflects the mobilization and production of various antioxidant molecules to preserve cellular integrity and organism function due to gabapentin-induced oxidative stress (Benzie and Strain 1996). An increase in FRAP value is usually desirable as it demonstrates better protection against damage due to reactive oxygen and nitrogen species (Hsieh and Rajashekaraiah 2021). In agreement with our findings, the increased antioxidant capacity measured by the FRAP assay has also been demonstrated in other scientific studies evaluating the effects of pharmaceuticals on fish (Mikula et al. 2024). The premise about the induction of oxidative stress after exposure to gabapentin is also supported by other significant findings, especially changes in antioxidant enzyme activities and an increase in lipoperoxidation. Antioxidant enzymes play a crucial role in combating oxidative stress in fish by neutralising reactive oxygen and nitrogen species and preventing cellular damage such as oxidation of proteins, DNA, or lipids (Martinez-Alvarez et al. 2005). Most significant alterations in the antioxidant enzyme activities were observed in the SOD, which is the first-line defence antioxidant and provides the conversion of superoxide radical to hydrogen peroxide (Martinez-Alvarez et al. 2005). Surprisingly, the opposite trend was found in the analysed tissues, with a significant increase in SOD activity in the liver but a reduction in the gill and kidney. The differential activity of SOD observed in analysed tissues suggests distinct tissue-specific responses to oxidative stress induced by this pharmaceutical. Increased SOD activity in the liver suggests an adaptive response to the detoxification due to gabapentin exposure. The liver, as the primary detoxification organ, increases its antioxidant defence to reduce the effects of reactive oxygen and nitrogen species generated during the biotransformation of gabapentin, which is, however, generally very limited in humans and animals (McLean 1994; Terry et al. 2010). In contrast, reduced SOD activity in the gill and caudal kidney suggests a different pattern. These organs are directly exposed to the environment (gill) and involved in the excretion process (caudal kidney), making them more susceptible to oxidative damage. Reduced SOD activity in these tissues may be a consequence of their exhaustion, which leads to a decrease in antioxidant defence and subsequent cell damage. The increase in lipid peroxidation in almost all treatment groups observed in the caudal kidney further supports the idea that oxidative damage is more pronounced in this organ, probably due to its role in the filtration and excretion of gabapentin. In addition, there was a statistically significant decrease in CAT and GST activities in the caudal kidney in the experimental group exposed to the highest concentration tested. These findings further support the hypothesis that due to the low biotransformation of gabapentin and its consequent increased excretion by the kidney in unchanged form, there is heightened stress on the kidney. This increased renal workload may lead to potential pathological changes, as evidenced by decreased antioxidant enzyme activities and increased lipid peroxidation observed in the caudal kidney tissues.

Other ecotoxicological studies confirmed the induction of oxidative stress following exposure to gabapentin. Li et al. (2018) investigated the toxicity of gabapentin in the early developmental stages of zebrafish (*Danio rerio*). They found that a 96-h exposure to the test substance at concentrations of 10 and 1 000 μg/l led to an increased GST, CAT and hydroxyl radical scavenging activity. Surprisingly, the elevation of CAT also occurred in the group exposed to the lowest environmentally relevant concentration, which was chosen to be the same as in our study, i.e., 0.1 μg/l. Furthermore, they showed that gabapentin exposure at concentrations of tens of mg/l caused an increased incidence of malformations. There was also an increase in heart rate and an increased incidence of behavioural disturbances. Behavioural changes were further demonstrated by Henry et al. (2022), who found that gabapentin at an environmentally relevant concentration of 400 ng/l, although not altering swimming activity of early developmental stages of zebrafish, significantly increased the organism’s activity to light stimuli, i.e., affecting photomotor activity. As already mentioned, the number of scientific papers dealing with the specific issue of the effects of gabapentin on fish is very limited. However, studies can be found that address the toxicity of other antiepileptic drugs, such as carbamazepine. Liang et al. (2022) studied the effects of carbamazepine on selected indices of oxidative stress in common carp. They confirmed that subchronic exposure to an environmentally relevant concentration (5 μg/l) for 28 days leads to significant changes in antioxidant and detoxification parameters indicative of oxidative damage to the organism. More extensive changes such as DNA or lipid damage were observed at even higher concentrations tested (50 and 100 μg/l).

Our findings are crucial for understanding the ecological risks associated with water pollution by gabapentin. They provide insights into how subchronic exposure to this pharmaceutical can affect vital physiological processes in fish, potentially leading to broader implications for fish health and aquatic ecosystem stability. By elucidating the biochemical and oxidative stress responses in carp, this research contributes to the growing body of knowledge required to inform about environmental risk assessments and help in developing regulatory policies aimed at mitigating the impact of pharmaceutical contaminants in aquatic environments. Our findings regarding gabapentin’s effects on kidney function highlight the potential risk of renal damage following chronic exposure to this drug. The low biotransformation rate of gabapentin results in its high renal excretion in unchanged form, placing increased oxidative stress on the kidney. This stress can lead to pathological changes and impaired kidney function, manifested in our study by increased activity of some plasma enzymes (e.g., CK), decreased activity of antioxidant enzymes in the caudal kidney, and increased lipid peroxidation in the caudal kidney.

The study provides preliminary evidence that exposure to gabapentin leads to disruption of metabolic pathways and induction of oxidative stress, even at low, environmentally relevant concentrations. Further research is needed to elucidate the mechanisms behind these biochemical and antioxidant responses and determine the long-term effects of gabapentin exposure on fish health and ecosystem dynamics. Understanding these pathways is crucial for developing strategies to mitigate the impact of pharmaceutical pollutants on non-target aquatic organisms such as fish.

## References

[R1] Baldissera MD, Baldisserotto B. Creatine kinase activity as an indicator of energetic impairment and tissue damage in fish: A review. Fishes. 2023 Jan;8:59.

[R2] Benzie IF, Strain JJ. The ferric reducing ability of plasma (FRAP) as a measure of “antioxidant power”: The FRAP assay. Anal Biochem. 1996 Jul 15;239(1):70-6. 8660627 10.1006/abio.1996.0292

[R3] Cahova J, Blahova J, Plhalova J, Marsalek P, Doubkova V, Hostovsky M, Divisova L, Mares J, Faggio C, Svobodova Z. Long-term exposure to polycyclic musk tonalide – A potential threat to juvenile zebrafish (Danio rerio)? Vet Med-Czech. 2023 May 29;68(5):218-24. 10.17221/40/2023-VETMED PMC10581524 37982026

[R4] Ferencik M, Blahova J, Schovankova J, Siroka Z, Svobodova Z, Kodes V, Stepankova K, Lakdawala P. Residues of selected anticonvulsive drugs in surface waters of the Elbe River Basin (Czech Republic). Water. 2022 Dec;14(24):4122.

[R5] Garg VK, Pandey A, Kataria N, Faggio C. Pharmaceuticals in aquatic environments – Toxicity, monitoring, and remediation technologies. Boca Raton (USA): CRC Press; 2023. 216 p.

[R6] Gomez Cortes L, Marinov D, Sanseverino I, Navarro Cuenca A, Niegowska Conforti M, Porcel Rodriguez E, Stefanelli F, Lettieri T. Selection of substances for the 4 Watch List under the Water Framework Directive. EUR 31148 EN. Luxembourg: Publications Office of the European Union; 2022. 101 p.

[R7] Gurke R, Roessler M, Marx C, Diamond S, Schubert S, Oertel R, Fauler J. Occurrence and removal of frequently prescribed pharmaceuticals and corresponding metabolites in wastewater of a sewage treatment plant. Sci Total Environ. 2015 Nov 15;532:762-70. 26124013 10.1016/j.scitotenv.2015.06.067

[R8] Haluzova I, Modra H, Blahova J, Marsalek P, Siroka Z, Groch L, Svobodova Z. Effects of subchronic exposure to Spartakus (prochloraz) on common carp Cyprinus carpio. Neuro Endocrinol Lett. 2010;31(Suppl 2):105-13. 21187831

[R9] He Y, Li X, Jia D, Zhang W, Zhang T, Yu Y, Xu Y, Zhang Y. A transcriptomics-based analysis of the toxicity mechanisms of gabapentin to zebrafish embryos at realistic environmental concentrations. Environ Pollut. 2019 Aug;251:746-55. 31121539 10.1016/j.envpol.2019.05.063

[R10] Henning N, Kunkel U, Wick A, Ternes T. Biotransformation of gabapentin in surface water matrices under different redox conditions and the occurrence of one major TP in the aquatic environment. Water Res. 2018 Jun;137:290-300. 29554533 10.1016/j.watres.2018.01.027

[R11] Henry J, Bai Y, Kreuder F, Saaristo M, Kaslin J, Wlodkowic D. Sensory-motor perturbations in larval zebrafish (Danio rerio) induced by exposure to low levels of neuroactive micropollutants during development. Int J Mol Sci. 2022 Aug 15;23(16):8990. 36012255 10.3390/ijms23168990 PMC9409309

[R12] Hoseinifar SH, Yousefi S, Doan HV, Ashouri G, Gioacchini G, Maradonna F, Carnevali O. Oxidative stress and antioxidant defense in fish: The implications of probiotic, prebiotic, and synbiotics. Rev Fish Sci Aquac. 2021 Jul;29(2):1-20.

[R13] Hsieh C, Rajashekaraiah V. Ferric reducing ability of plasma: A potential oxidative stress marker in stored plasma. Acta Haematol Pol. 2021 Jan;52(1):61-7.

[R14] Li X, Zhou S, Qian Y, Xu Z, Yu Y, Xu Y, He Y, Zhang Y. The assessment of the eco-toxicological effect of gabapentin on early development of zebrafish and its antioxidant system. RSC Adv. 2018 Jun 7;8(40):22777-84. 35539713 10.1039/c8ra04250k PMC9081491

[R15] Liang Y, Csenki Z, Ivanovic B, Bock I, Csorbai B, Molnar J, Vasarhelyi E, Griffitts J, Ferincz A, Urbanyi B, Acs A. Biochemical marker assessment of chronic carbamazepine exposure at environmentally relevant concentrations in juvenile common carp (Cyprinus carpio). Antioxidants. 2022 Jun 6;11(6):1136. 35740033 10.3390/antiox11061136 PMC9219654

[R16] Martinez-Alvarez RM, Morales AE, Sanz A. Antioxidant defences in fish: Biotic and abiotic factors. Rev Fish Biol Fish. 2005 Feb;15:75-88.

[R17] Martinez-Porchas M, Martinez-Cordova LR, Ramos-Enriquez R. Cortisol and glucose: Reliable indicators of fish stress. Pan-Am J Aquat Sci. 2009 Feb;4(2):158-78.

[R18] Mikula P, Hollerova A, Hodkovicova N, Doubkova V, Marsalek P, Franc A, Sedlackova L, Hesova R, Modra H, Svobodova Z, Blahova J. Long-term dietary exposure to the non-steroidal anti-inflammatory drugs diclofenac and ibuprofen can affect the physiology of common carp (Cyprinus carpio) on multiple levels, even at „environmentally relevant“ concentrations. Sci Total Environ. 2024 Mar 20;917:170296. 38301789 10.1016/j.scitotenv.2024.170296

[R19] Morasch B, Bonvin F, Reiser H, Grandjean D, de Alencastro LF, Perazzolo C, Chevre N, Kohn T. Occurrence and fate of micropollutants in the Vidy Bay of Lake Geneva, Switzerland. Part II: Micropollutant removal between wastewater and raw drinking water. Environ Toxicol Chem. 2010 Aug;29(8):1658-68. 20821617 10.1002/etc.222

[R20] O’Rourke K, Engelmann B, Altenburger R, Rolle-Kampczyk U, Grintzalis K. Molecular responses of daphnids to chronic exposures to pharmaceuticals. Int J Mol Sci. 2023 Feb 17;24(4):4100. 36835510 10.3390/ijms24044100 PMC9964447

[R21] Nolasco-Soria H. Amylase quantification in aquaculture fish studies: A revision of most used procedures and presentation of a new practical protocol for its assessment. Aquaculture. 2021 May 1;538:736536.

[R22] Salahinejad A, Meuthen D, Attaran A, Chivers DP, Ferrari MCO. Effect of common antiepileptic drugs on teleost fishes. Sci Total Environ. 2023 Mar 25;866:161324. 36608821 10.1016/j.scitotenv.2022.161324

[R23] UNESCO, HELCOM. Pharmaceuticals in the aquatic environment of the Baltic Sea region – A status report. UNESCO Emerging Pollutants in Water Series – No. 1, Paris: UNESCO Publishing; 2017. 121 p.

[R24] McLean MJ. Clinical pharmacokinetics of gabapentin. Neurology. 1994 Jun;44(6 Suppl 5):S17-S22. 8022536

[R25] Tamber SS, Bansal P, Sharma S, Singh RB, Sharma R. Biomarkers of liver diseases. Mol Biol Rep. 2023 Sep;50(9):7815-23. 37482588 10.1007/s11033-023-08666-0

[R26] Terry RL, McDonnell SM, van Eps AW, Soma LR, Liu Y, Uboh CE, Moate PJ, Driessen B. Pharmacokinetic profile and behavioral effects of gabapentin in the horse. J Vet Pharmacol Therap. 2010 Oct;33(5):485-94. 20840393 10.1111/j.1365-2885.2010.01161.x

